# The Effects of Virtual Immersive Gaming to Optimize Recovery (VIGOR) in Low Back Pain: A Phase II Randomized Controlled Trial

**DOI:** 10.3390/healthcare14020142

**Published:** 2026-01-06

**Authors:** Susanne M. van der Veen, Alexander Stamenkovic, Christopher R. France, Amanda Robinson, Roy Sabo, Forough Abtahi, James S. Thomas

**Affiliations:** 1 Physical Therapy, College of Allied Health Sciences, East Carolina University, Greenville, NC 27858, USA; 2 Human Factors & User Experience, J.S. Held LLC, Redmond, WA 98052, USA; alexander.stamenkovic@jsheld.com; 3 Psychology, College of Arts and Sciences, Ohio University, Athens, OH 45701, USA; france@ohio.edu; 4 Wright Center, Virginia Commonwealth University, Richmond, VA 23298, USA; robinsonam7@vcu.edu; 5 School of Public Health, Biostatistics, Virginia Commonwealth University, Richmond, VA 23219, USA; rsabo@vcu.edu; 6 Physical Therapy, College of Health Professions, Virginia Commonwealth University, Richmond, VA 23220, USA; abtahinezhas@vcu.edu (F.A.); jthomas32@vcu.edu (J.S.T.)

**Keywords:** virtual reality, low back pain, kinesiophobia, clinical trial

## Abstract

**Highlights:**

**What are the main findings?**

- Pain and disability can be reduced by inducing people with chronic low back pain and fear of movement to bend their back.
- The reductions in pain and disability were maintained over the 48-week follow-up.

**What is the implication of the main finding?**

- Treatment of kinesiophobia and/or chronic low back pain could benefit from gamification and gradually inducing lumbar flexion movements.

**Abstract:**

**Background:** Chronic low back pain (cLBP) with kinesiophobia is difficult to treat, and traditional graded activity approaches often show limited adherence and short-term effects. Virtual reality (VR) may enhance treatment engagement by providing immersive game-based environments that encourage therapeutic movement. This randomized controlled trial aimed to examine the effects of VR interventions designed to promote lumbar spine flexion in individuals with cLBP and elevated movement-related fear. **Methods:** Participants were randomized to one of two nine-week VR game conditions that differed only in the amount of lumbar flexion required. Primary outcomes were changes in pain intensity and disability from baseline to one-week post-treatment. Secondary analyses examined lumbar flexion and expectations of pain/harm as potential mediators. Follow-up assessments were conducted at multiple time points through 48 weeks to assess maintenance of treatment gains. **Results:** Both VR groups showed significant and clinically meaningful reductions in pain and disability at post-treatment. Improvements were maintained throughout the 48-week follow-up period. Depression symptoms continued to improve during follow-up. Expectations of pain and harm decreased significantly during treatment and remained reduced, whereas objective lumbar flexion did not change appreciably over time. Mediator analyses indicated that improved expectations of pain/harm, rather than increased lumbar flexion, were more closely associated with treatment response. **Conclusions:** Immersive VR gaming produced sustained reductions in pain, disability, and movement-related fear in individuals with cLBP and kinesiophobia. Findings suggest that VR may enhance rehabilitation outcomes by modifying maladaptive expectations rather than altering lumbar motion. VR-based interventions represent a promising and engaging approach for long-term cLBP management.

## 1. Introduction

Kinesiophobia—defined as a fear of movement driven by expectations of pain or injury—is one of the strongest predictors of chronic low back pain (cLBP) development [1,2,3,4]. Although avoiding movement may temporarily ease anxiety in individuals with back pain, prolonged reductions in activity can lead to the shortening of spinal peri-articular connective tissues, alterations in the surrounding musculature [5,6,7], and an increased likelihood of the pain becoming chronic.

Common cLBP treatment strategies include graded activity and graded exposure therapies. Graded activity aims to restore the functional ability independent of pain levels by highlighting the benefits of physical activity and the detrimental effects of inactivity on overall health. This approach typically uses predetermined activity quotas and rewards incremental increases in activity over time. In contrast, graded exposure targets the reduction in fear, pain, and harm expectations that accompany movement, emphasizing fear as a barrier to recovery. This method involves constructing a personalized hierarchy of feared movements, which then serves as the basis for systematic exposure and opportunities to challenge inaccurate expectations of harm. Although randomized controlled trials show that both approaches can significantly reduce pain and disability [8,9], a systematic review found that graded activity was not superior to other exercise-based treatments and that graded exposure offers no advantage over wait-list or usual-care controls [10]. While several studies have demonstrated that VR interventions can produce short-term reductions in pain intensity, disability, and kinesiophobia compared with minimal or usual-care controls, evidence supporting the sustained long-term benefits remains limited [11,12,13]. Notably, most of these interventions were designed to influence pain and movement behavior through non-specific mechanisms, such as attentional distraction, altered sensory feedback, and increased motivation for movement, rather than by directly targeting specific motor behaviors or movement patterns [11,13]. One explanation for these limited effects is that patients may perform the prescribed tasks without increasing the lumbar flexion. Our previous work demonstrates that pain-related fear is consistently associated with limited lumbar flexion in people with subacute LBP [14,15], those with cLBP [16], individuals recently recovered from LBP [17], and even healthy individuals experiencing experimentally induced back pain [15]. Thus, a persistent limitation of lumbar motion may hinder optimal functional recovery.

To address this issue, we created virtual reality games designed to progressively increase lumbar flexion as part of gameplay. These games do not explicitly target psychological factors underlying avoidance; instead, they provide acute distraction from pain, positive reinforcement for movement, and gradual increases in lumbar flexion demands. In a Phase I clinical trial [18], we found that three daily sessions of virtual dodgeball were safe for individuals with cLBP, did not worsen their pain, were positively received, and elicited increased lumbar flexion during play. Building on these results, we developed a Phase II randomized controlled trial: a nine-week intervention known as Virtual Immersive Gaming to Optimize Recovery (VIGOR) [19].

## 2. Materials and Methods

**Figure 1:**
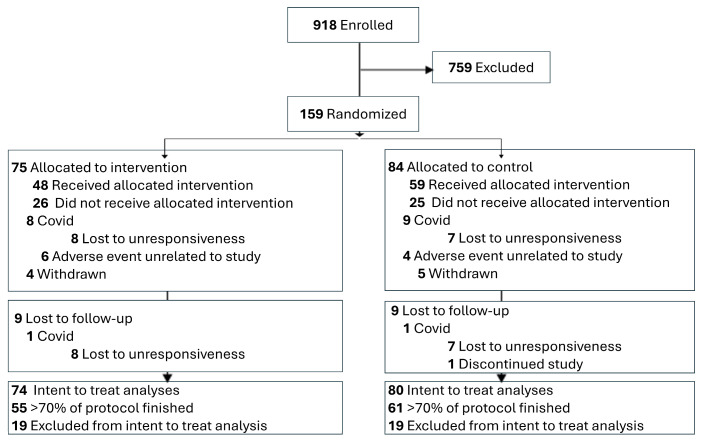
Consort diagram of participant enrollment, allocation, follow-up, and analysis.

This randomized clinical trial was a triple-blinded (participants, researchers, and analysts were blinded) 2-arm trial. The Institutional Review Board of Virginia Commonwealth University approved this study (HM20014058, 22 October 2018), and informed consent was obtained from all participants. The detailed study protocol is published [19] and registered at the National Library of Medicine (NCT03463824, 13 February 2018). Recruitment began on 10 January 2019, the primary completion date was 17 January 2023, and the study completion date was 12 December 2023. Participants were randomized in a 1:1 ratio into 1 of 2 groups (Figure 1).

Those assigned to the experimental group played our immersive video games that encouraged participants to produce progressively larger lumbar flexion excursions at each game level and across treatment sessions, i.e., virtual balls were launched to elicit 15°, 30°, 45°, and 60° [19]. Those assigned to the control group played the same immersive games, but the parameters were modified such that less lumbar flexion was required to achieve the same game objectives, i.e., virtual balls were launched to elicit −15°, 0°, 15°, and 30°. Both participants and administrators of the VR game were blinded to the group assigned, as the participant identification code was entered in the VR menu and linked to the randomization table controlling the target heights of the virtually launched balls, as explained below. The treatment frequency and duration were based on prior research on graded activity and exposure, typically with 6–12 interventions with 8–18 treatment sessions, resulting in significant reductions in disability [10,20]. Accordingly, participants in this study completed 18 intervention visits over 9-weeks with the number of sessions tapered across weeks (i.e., 3 sessions/week in weeks 1–3, 2 sessions/week in weeks 4–6, and 1 session/week in weeks 7–9, See Table 1). Our co-primary outcome variables were change in pain and change in disability from baseline to 1-week post-treatment (Aim 1). We also examined changes in expectations of pain/harm and lumbar flexion as potential mechanisms of a change in pain and disability (Aim 2). Finally, we examined the maintenance of treatment gains at 1, 6, 12, 24, and 48 weeks post-treatment (Aim 3).

### 2.1. Aims and Hypotheses

#### 2.1.1. Aim 1

Examine immediate clinical outcomes as a function of treatment. We hypothesized that, relative to the control group, participants in the experimental group would show larger reductions in pain and disability at post-treatment relative to pre-treatment baseline values (Hypothesis 1).

#### 2.1.2. Aim 2

Examine potential mechanisms of pre- to post-treatment changes in clinical outcomes. We hypothesized that participants in the experimental group would exhibit larger pre- to post-treatment decreases in pain/harm expectancy and increases in lumbar flexion as compared to the control group (Hypothesis 2.1). We further posited that decreases in pain/harm expectancy and increases in lumbar flexion would be positively related to pre- to post-treatment reductions in pain and disability (Hypothesis 2.2).

#### 2.1.3. Aim 3

Examine maintenance of treatment gains. We hypothesized that, relative to the control group, participants in the experimental group would continue to show lower levels of pain and disability at 1, 6, 12, 24, and 48 weeks after the last treatment session (Hypothesis 3).

### 2.2. Recruitment and Pre-Screening

People with low back pain were recruited through advertisements and flyers posted in the local community and via a combination of electronic, radio, and print. We also recruited from local clinics and ResearchMatch.org. This recruitment resulted in 918 people completing a brief online survey using REDCap to determine their initial eligibility [21]. This pre-screening survey covered the main inclusion and exclusion criteria (see Table 1), including a numeric pain rating scale (24 h and 7-day recall), the Roland–Morris Disability Questionnaire [22,23], a question about fear of physical activity, and medical history related to back pain. In general, a majority of the potential participants were excluded due to (1) insufficient pain levels, (2) insufficient duration of symptoms, (3) insufficient kinesiophobia levels (i.e., tsk < 37), (4) age outside of criterion. Individuals who met the initial study criteria (n = 220) were scheduled for an in-person physical screening prior to assignment to a treatment group.

**Table 1:** Participant inclusion and exclusion criteria.

Inclusion criteria
18–60 years of age Low back pain for at least half of the days in the past 6 months Average pain intensity > 3 on a 0–10 numeric pain rating scale Disability > 4 on Roland and Morris Disability Kinesiophobia > 36 Tampa scale for kinesiophobia Agrees with the statement “It is not really safe for a person with my back problem to be physically active“ Has sought care or consultation from a healthcare provider for back pain Meets < 4 on the Classification System Quebec Task Force on Spinal Disorders [24], reflecting low back pain without neurological signs. Working proficient English
Exclusion criteria
A personal history of the following neurological disorders: Alzheimer’s, amyotrophic lateral sclerosis, multiple sclerosis, Parkinson’s, neuropathy, stroke, seizures A personal history of the following cardiorespiratory disorders: congestive heart failure, heart attack in the past 2 years A personal history of the following musculoskeletal disorders: rheumatoid arthritis, muscular dystrophy, pathologic fractures of the spine, avascular necrosis or osteonecrosis, severe osteoarthritis
A personal history of spine surgery or hip arthroplasty Active cancer A chronic disease that may restrict movement or preclude safe participation Used opioids or muscle relaxants within 30 days prior to study enrollment Reports being pregnant, lactating, or that they anticipate becoming pregnant within 2 months Reports pending litigation related to the cLBP Current drug or alcohol use, which in the opinion of the PIs would interfere with the adherence to study requirements Significant visual impairment that would prevent virtual reality headset use
Significant motion sickness that would prevent virtual reality use

### 2.3. Screening, Consent, and Enrollment (Visit 0)

During the screening session, the study protocol was verbally described to candidates, any questions were answered, and the informed consent process was started in a quiet private room. Participants were shown a video of the virtual reality games to help them with the process. If they wished to continue, they were asked to read and sign an informed consent document. Those who provided informed consent (N = 220) then completed a series of screening surveys (see Table 2), including a repeat of those completed as part of the pre-screening.

Additionally, individuals who continued to meet the inclusion/exclusion criteria (N = 159, N = 61 were lost due to a reduced TSK scored < 37 during screening) underwent a physical exam by a physical therapist. Participants who remained eligible following the physical exam were formally enrolled into the study (N = 159). They were randomly assigned into a treatment group using a randomization table created by the study statistician prior to study onset. They then proceeded to a pre-treatment baseline assessment (visit 1), which occurred within seven days of the screening visit (or else the participants were re-screened).

### 2.4. Pre-Treatment and Baseline Assessment (Visit 1)

The pre-treatment baseline assessment included a series of survey measures, participation in a standardized reaching task, and real-world activity monitoring. As shown in Table 2, the survey measures include numeric pain rating scales (current, 24 h, and 7-day recall), the Roland–Morris Disability Questionnaire [22,24], a medication log, and a range of psychological measures (e.g., Tampa Scale for Kinesiophobia [25], Center for Epidemiologic Studies—Depression [26], Pain Catastrophizing Scale [27], Pain Resilience Scale [28,29]).

**Figure 2:**
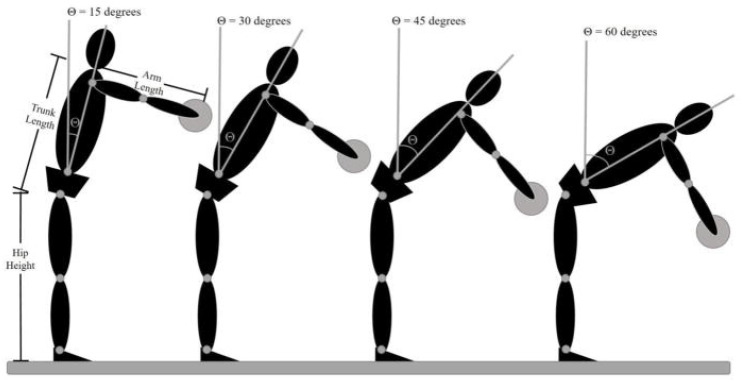
Target locations determined for each individual participant based on hip height, trunk length, and arm length. The high target was located such that the subject could, in theory, reach the target by flexing the hips 15° with the shoulder flexed 90° and the elbow extended. Published by France and Thomas 2018 [19].

For the standardized reaching task, participants wore an HMD (head-mounted display) and touched virtual targets, while the movement of light-reflective marker clusters attached to their head, upper arms, forearms, hands, trunk, pelvis, thighs, shanks, and feet were recorded using a 12-camera Vicon Bonita system (Vicon Motion Systems Ltd., Oxford, UK). Participants touched 4 virtual targets co-located in the mid-sagittal plane. As shown in Figure 2, the target locations were adjusted to participant anthropometrics to allow for the comparison of movement patterns across individuals in a task that requires progressive increases in lumbar spine flexion [30] Participants performed ten reach trials for each virtual target location: five per hand, pausing at the target for two seconds, then returning to an upright posture. Instructions emphasized that participants should reach for the targets as quickly as possible in a way that is “natural and comfortable for them.” This instruction was used to avoid biasing participants with a perceived correct way to move and a rapid pace that would challenge the participant by increasing the loading required to perform the task. While forward excursions of the trunk must be counter-balanced by backward movement of the lower extremities, the targets were located such that they did not require an individual to move anywhere near the limits of the available range of motion of the lumbar spine, pelvis, knee, and ankle. Thus, participants could reach the targets using an infinite combination of joint excursions. Even though the reaching task required no lifting, and the loads on the lumbar spine were small, we have shown that individuals with elevated levels of kinesiophobia exhibit reduced lumbar spine flexion at this combination of target height and reaching speed [14,16,17,30]. The time series joint angle data were calculated from the 3D segment coordinate data using a Euler angle sequence of: (1) flexion–extension, (2) lateral bending, and (3) axial rotation using Motion Monitor software 4.0 (MotionMonitor xGEN, Innovative Sports Training Inc., Chicago, IL, USA) [31]. The standardized reaching paradigm was used to assess three dependent measures associated with Aim 2 (i.e., lumbar flexion and pain/harm expectancy). Lumbar flexion was defined as the change in joint angle during each reach (i.e., the difference between the joint angle at the beginning of the trial before the go signal and the joint angle recorded 100 ms after target contact). Consistent with our prior work [18,32,33,34], expectations of pain and harm were also measured during standardized reaches. For each target height, prior to the first reaching trial, participants rated the level of “expected pain” and “expected harm” using a visual analog scale displayed through the head mounted display. The scale consisted of a 10 cm horizontal line with no numbers, marks, or descriptive vocabulary along its length. For expected pain ratings, the scale was anchored with the descriptors “No pain” and “Worst pain imaginable”, respectively, at each end of the line. For expected harm, the scale was anchored with “Not at all concerned” and “Extremely concerned” regarding potential harm to the back during task performance. Participants indicated their response by moving a virtual sliding scale.

After completing all indicated pre-treatment assessments, participants viewed a brief (11.5 min) treatment rationale video that included a chronic pain educational component designed to explain how pain persists without underlying pathology and to describe the interaction between biological, psychological, and social factors in maintaining chronic back pain and related disability. Following the treatment rationale video, participants were introduced to the virtual video games for the first time and had an opportunity to play a practice level to review the basic game constructs (e.g., scoring metrics, moving the avatar, sound and visual cues). They then completed the Treatment Evaluation Inventory to assess their perceptions and expectations regarding the proposed treatment.

### 2.5. Treatment (Visits 2–19)

**Figure 3:**
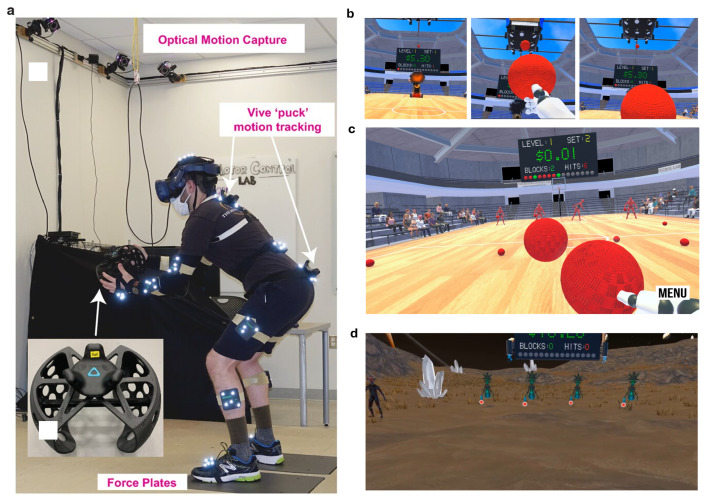
A participant with the head-mounted display and instrumented with the marker clusters (**a**). Dodgeball variants are illustrated: cannon (**b**), standard (**c**), alien planet (**d**).

As shown in Table 3, participants completed 18 intervention visits over 9 weeks with the number of sessions tapered across weeks (i.e., 3 sessions/week in weeks 1–3, 2 sessions/week in weeks 4–6, and 1 session/week in weeks 7–9). The immersive games varied across the 9 weeks of treatment to provide a graded increase in challenge with respect to lumbar spine motion, encourage player engagement, and prevent player boredom. There were four virtual reality environments, each played from a first-person perspective, including Dodgeball Cannon, Day, Night, and Space (see Figure 3).

All *Dodgeality* versions consisted of launched virtual balls that were directed at the participant’s avatar. The goal was to either block the launched virtual ball with a virtual ball held by the participant’s avatar or to duck the launched virtual ball if it changed color and was accompanied by a quacking sound. In all variants of *Dodgeality*, there were 15 launched virtual balls per set, 2 sets per level, and 3 levels per game. Performance was updated in real time and displayed on a virtual scoreboard. In week one, participants played three sessions of a version of the game, where the balls were launched in slow arcs by a cannon located inside an arena (i.e., Dodgeball Cannon). This was the least physically challenging version of *Dodgeality*, as the balls started from one location, and the launch trajectory had a high parabolic flight pattern to make interception of the launched virtual ball less challenging. In week two and the beginning of week three, participants played four sessions of a traditional dodgeball game within the same virtual arena environment (i.e., Dodgeball Day). In this version, they competed against four opponent avatars who randomly took turns throwing balls using a normal human motion. In weeks three, four, and five, participants played five sessions of the same traditional dodgeball game, except that the arena lights were removed, and the opposing avatars launched virtual glow-in-the-dark balls (i.e., Dodgeball Night). Finally, in weeks five through nine, participants played weekly sessions of traditional dodgeball that took place on the surface of an alien planet (i.e., Dodgeball Space). The opposing players were aliens, and the physics of the ball launches were adjusted to reflect the reduced gravity of the moon. During weeks five through nine, the launch velocity of virtual balls increased by 10% if success was higher than 80%. Thus, the initial launch velocity increased from 45 m/s to 49.5 m/s if over 24 out of 30 balls were blocked that day. Progressively increasing the initial launch velocity necessitated more rapid movements of the participants to successfully intercept the launched virtual balls and thereby increased the difficulty of the tasks. See Table 3 for the game allocation per visit.

In the control group, to ensure that lumbar flexion was minimized while playing the virtual reality games, we manipulated the presentation of the virtual targets such that the participant only needed to flex the spine 0–45° to successfully intersect the virtual object instead of 15–60° the treatment group.

As shown in Table 2, the survey measures taken during every treatment visit included a numeric pain rating for current pain, medication log, and adverse events. During every first visit of the week (Visit 2, 5, 8, 11, 13, 15, 17, 18, and 19), additional numeric pain rating scales for 24 h and 7-day averages and a standardized reaching task as described in the pre-treatment were completed.

### 2.6. Post-Treatment Follow-Up Assessments (Visits 20–24)

After the participants completed 18 game sessions over nine weeks, post-treatment follow-up sessions (visits 20–24) were scheduled to assess the maintenance of treatment gains at 1, 6, 12, 24, and 48 weeks after the last treatment session. These follow-up sessions were identical to the pre-treatment baseline assessments with two exceptions. First, at each of the follow-up visits, participants completed a one-item Patient Global Impression of Change measure [35] to assess their overall sense of improvement as a function of receiving the treatment. Second, participants repeated the Treatment Evaluation Inventory [36] to assess their acceptance of the virtual reality games as a potential intervention for low back pain at the first follow-up session only (i.e., visit 20).

### 2.7. Additional Design Points

To monitor the safety, participants completed a brief health screening at the beginning of each game session to determine whether there were any changes in back pain or radiating symptoms. Any negative change in health status, other than back pain, was recorded as an adverse event, logged, and reported per the requirements of the Virginia Commonwealth University IRB. In the case of deviation from the protocol due to negative health changes, the study’s safety committee would meet to determine whether the adverse event was caused by the intervention.

Participants discontinued the study intervention when a medical condition developed that precluded the continuation of the treatment intervention. If participants discontinued prior to completing all scheduled treatment sessions (regardless of the reason), we made every attempt to obtain the outcome measurements. If the study participant was unwilling or unable to undergo the laboratory-based tests, we still attempted to obtain the clinical outcome measures. In instances where an adverse event occurred, we followed-up with the participant until the event was resolved or until the IRB deemed it unnecessary to continue to follow the participant.

All participants in active treatment during the COVID-19 pandemic (between 12 March and 26 July 2020, N = 17) were withdrawn from the study. Participants who were already in the follow-up phase of the study did not do a standardized reaching task but finished the surveys online (number of online surveys per follow-up visit; v20 = 0, v21 = 3, v22 = 7, v23 = 19, v24 = 14, participant N = 34); however, some participants did not finish the online surveys (N = 3). When the lab opened back up for in-person visits on 27 July 2020, participants were encouraged to come back for their follow-up assessments; however, one participant chose to complete their last follow-up visit (v24) online.

### 2.8. Outcomes, Sample Size Calculations, and Analysis

We were interested in several outcomes in this study: current, 24 h, and 7-day recall pain (NPRS scale); the Roland–Morris disability score (RMDQ); expectation of pain or harm as measured by the Tampa scale for kinesiophobia (TSK); depression as measured by the eight item PROMIS-depression scale; pain catastrophizing (PCS); Global Impression of Change (GIC); and lumbar flexion angles measured to targets positioned to elicit 15, 30, 45, and 60 degrees of lumbar flexion (respectively levels 1, 2, 3, and 4) as described in the baseline section. Outcomes were collected pre-treatment as well as at 1, 6, 12, 24, and 48-weeks post-treatment, except for the Global Impression of Change, which was only assessed at post-treatment. Study data were collected and managed using REDCap electronic data capture tools hosted by Virginia Commonwealth University [21].

#### 2.8.1. Statistical Analysis

Sample size calculations for this study have been previously described [19]. In brief, based on extant studies of pain (0–10 NPRS scale) and disability (RMDQ), we used a Cohen’s d effect size of 0.45 for simulation studies that indicated 230 subjects would be sufficient to achieve power of 0.8 to detect a clinically meaningful treatment effect for outcomes at our primary end point (v20). The sample size calculations also assumed a 10% attrition rate.

The primary hypotheses were that (1) the treatment group (game designed to elicit 15–60 degrees lumbar flexion) would display a significantly larger reduction in NPRS and RMDQ levels from baseline than the control (game designed to elicit 0–45 degrees lumbar flexion) at the primary end point, (2) participants in the treatment group would exhibit larger pre- to post-treatment decreases in kinesiophobia (TSK) and pain catastrophizing (PCS) and increases in lumbar flexion, as compared to the control group, and (3) relative to the control group, participants in the treatment group would continue to show lower levels of pain (NPRS) and disability (RMDQ) at each follow-up time point.

Data are summarized overall and by treatment group. Numerical measurements are summarized with the mean and standard deviation. Categorical measurements are summarized with frequencies and percentages. The total number of missing responses are reported for each measure.

Linear mixed-effects regression analyses served as the main analytic framework. The maximum-likelihood estimation allowed participants with missing data to be included in the model if the observations were missing completely at random or missing at random. See Appendix A. Mixed effect model outcome table.

All regression models incorporated random intercept terms to account for within-subject variance associated with longitudinal experimental designs. We compared models with linear and quadratic time using likelihood ratio tests and included time as a quadratic term when appropriate due to potential nonlinear relationships between outcomes and time. The fixed effects included the group, linear time (with baseline as the reference), quadratic time when indicated, and their interaction terms (group × time and group × time^2^) to evaluate group differences in both linear and nonlinear change over time. Biological sex, BMI, and radiating pain were included in each model as additional fixed effects. A priori simple interaction contrasts were conducted to compare the changes from baseline between the two intervention groups. All analyses were completed in R 4.5.0.

#### 2.8.2. Intention-to-Treat Analysis

All randomized study participants with at least a baseline assessment and complete records were included in the intention to-treat (ITT) analysis. This trial enrolled and randomized participants following an in-person physical examination. The baseline measures were then completed in a follow-up visit within 7 days of physical screening. Five of the randomized participants dropped out before baseline measures. Accordingly, our primary analysis does not meet the strict definition for ITT analysis. Thus, we used a near ITT analysis as the primary method for assessing outcomes, and maximum-likelihood estimation was used to estimate any missing observations.

#### 2.8.3. Per-Protocol Analysis

For the per-protocol analysis (PPA), we excluded study participants who (1) attended fewer than 13 treatment sessions and (2) received prohibited concomitant interventions or (3) developed an exclusionary medical condition while on the study protocol. The outcomes from the ITT analysis and PPA were nearly identical (i.e., the differences in estimated marginal means were generally less than 0.5, with no differences in significance). Accordingly, only the results of the ITT analysis are presented.

## 3. Results

A total of 159 participants (101 women [66%] and 53 men [34%]) with a mean (SD) age of 39.5 (12.9) years (range, 18.1–60.9 years) were enrolled in this trial. At baseline, this cohort reported a mean (SD) pain score over the last 7 days of 5.7 (1.9) on the NPRS (range, 0–10), a mean (SD) score of 13.2 (5.4) on the Roland–Morris Disability Questionnaire (range, 0–24), and a mean (SD) length of chronic back pain of 10.5 (10.2) years, indicating moderate back pain of long duration. Seventy-four participants were randomized to the treatment group. Five participants withdrew from the study after the randomization process owing to scheduling issues (one from the treatment and four from the control group) and never completed baseline measures or any part of the treatments and follow-up assessments. An additional 119 individuals did not complete the allocated intervention and follow-up visits. Participant characteristics as a function of the treatment group are reported in Table 4.

### 3.1. Primary Outcomes

#### 3.1.1. Treatment Effect on Pain and Disability

At the primary end point, there was no significant difference in the change in pain scores between the treatment and control groups for current pain (0.31; 95% CI, −0.51 to 1.13; *p* = 0.86), pain over the last 24 h (0.49; 95% CI, −0.35 to 1.33; *p* = 0.51), and pain over the last 7-days (0.37; 95% CI, −0.49 to 1.22; *p* = 0.80). There was no significant difference in the change in self-reported disability scores between the treatment and control (−0.13; 95% CI, −2.53 to 2.27; *p* = 1.00).

**Figure 4:**
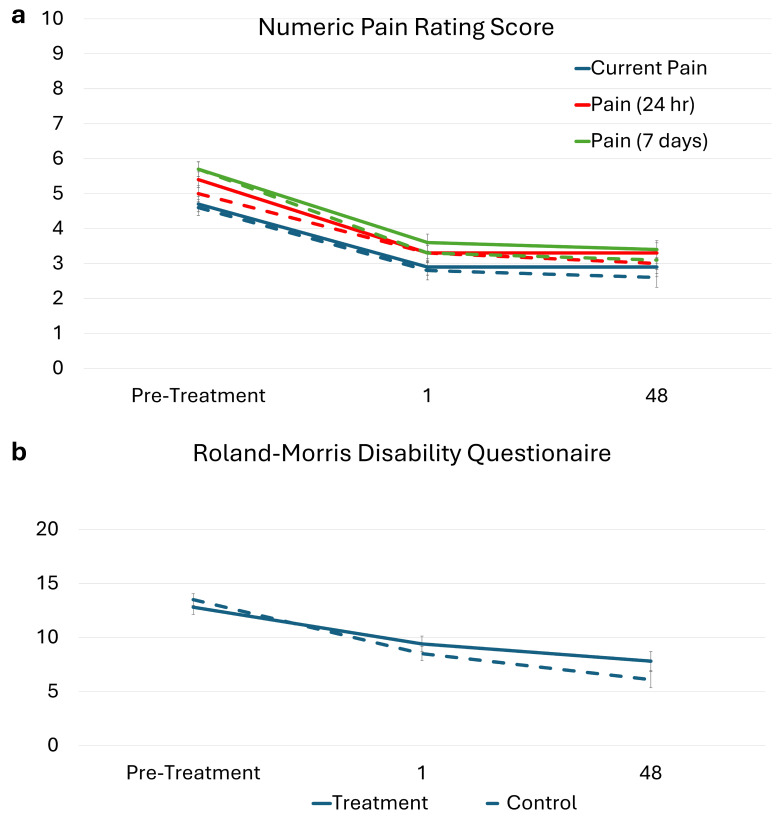
(**a**) Self-reported assessment of current pain, average pain over 24 h, and average pain over the last 7 days (Numerical Pain Rating Scale score; range, 0–10, with higher scores indicating more pain). Solid line represents the treatment and dashed line the control group. (**b**) Self-reported disability (Roland–Morris Disability Questionnaire; range, 0–24, with higher scores indicating higher disability). Standard error of the mean (error bars) is plotted for ratings collected at the initial visit, 1 week after completing 18 weeks of treatment (i.e., primary end point), and 48 weeks after treatment completion (i.e., follow-up). Solid lines represents the treatment and dashed line the control group.

There were significant changes in current pain at the primary end point within the treatment (−0.75; 95% CI, −1.10 to −0.39; *p* < 0.001) and control group (−1.03; 95% CI, −1.37 to −0.69; *p* < 0.001), pain over 24 h at the primary end point within the treatment (−0.85; 95% CI, −1.21 to −0.49; *p* < 0.001) and control group (−1.13; 95% CI, −1.47 to −0.80; *p* < 0.001), and pain over 7 days at the primary end point within the treatment (−0.91; 95% CI, −1.25 to −0.56; *p* < 0.001) and control group (−1.23; 95% CI, −1.56 to −0.91; *p* < 0.001). See Table 5 and Figure 4.

#### 3.1.2. Treatment Effect on Pain and Harm Expectancy and Lumbar Flexion Angles

At the primary end point, there was no significant difference in the change in kinesiophobia scores between the treatment and control groups (1.26; 95% CI, −1.36 to 3.88; *p* = 0.71) or pain catastrophizing (0.81; 95% CI, −3.74 to 5.36; *p* = 0.99). There were no significant differences in the change in lumbar flexion angles between the treatment and control groups for height 1 (left (−0.75; 95% CI, −3.69 to 2.20; *p* = 0.91), right (−0.59; 95% CI, −3.09 to 1.91; *p* = 0.92)), height 2 (left (1.26; 95% CI, −3.90 to 6.41; *p* = 0.97), right (−0.57; 95% CI, −4.85 to 3.70; *p* = 0.9.98)), height 3 (left (0.08; 95% CI, −5.42 to 5.58; *p* = 1.00), right (−0.26; 95% CI, −5.58 to 5.06; *p* = 0.99)), and height 4 (left (−1.25; 95% CI, −6.99 to 4.50; *p* = 0.94), right (−0.17; 95% CI, −6.01 to 5.68; *p* = 1.00)).

**Figure 5:**
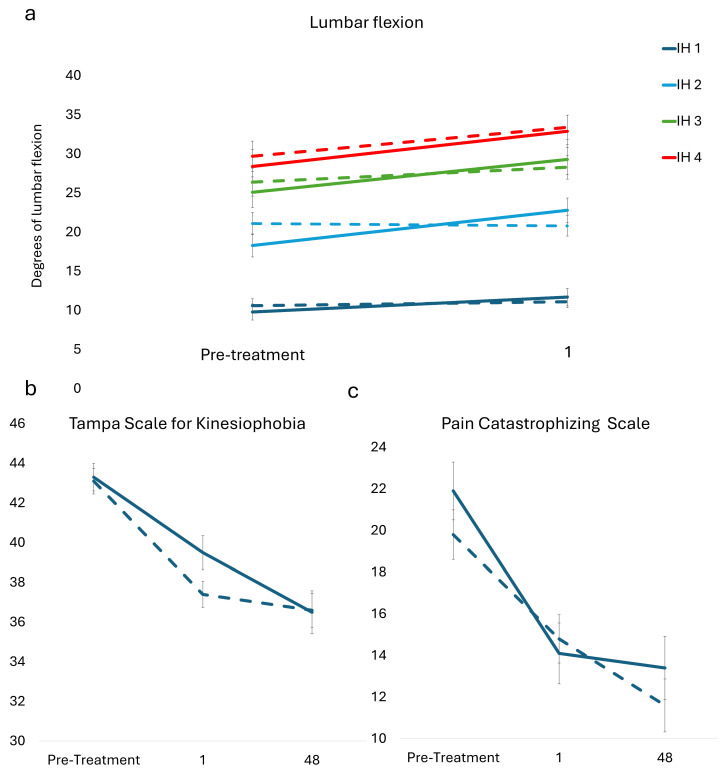
(**a**) Lumbar flexion angles to targets positioned to elicit 15° (dark blue), 30° (light blue), 45° (green), and 60° (red) lumbar flexion from pre-treatment to one week follow-up. Self-reported outcomes for (**b**) Tampa Scale for Kinesiophobia, and (**c**) Pain Catastrophizing Scale. Standard error of the mean (error bars) is plotted for ratings collected at the initial visit, 1 week after completing 18 weeks of treatment (i.e., primary end point), and 48 weeks after treatment completion (i.e., follow-up). Solid lines represent the treatment group and dashed lines the control group.

There were significant changes in kinesiophobia at the primary end point within the treatment (−2.81; 95% CI, −3.75 to −1.86; *p* < 0.001) and control groups (−3.20; 95% CI, −4.09 to −2.31; *p* < 0.001) and pain catastrophizing at the primary end point within the treatment (−4.48; 95% CI, −6.06 to −2.91; *p* < 0.001) and control groups (−3.15; 95% CI, −4.65 to −1.66; *p* < 0.001). See Table 5 and Figure 5.

#### 3.1.3. Maintenance of Treatment Gains Post-Treatment

The maintenance of the treatment gains (48 weeks post-treatment) were not significant different in the change in pain scores between the treatment and control groups for current pain (0.36; 95% CI, −0.77 to 1.49; *p* = 0.93), pain over the last 24 h (0.40; 95% CI, −0.74 to 1.55; *p* = 0.90), or pain over the last 7-days (0.34; 95% CI, −0.79 to 1.47; *p* = 0.95). There was no significant difference in the change in self-reported disability scores between the treatment and control groups (0.85; 95% CI, −2.05 to 3.76; *p* = 0.95). For treatment gains at 6, 12, 24, and 48 weeks post treatment, see the table in Appendix B).

There were significant changes in the current pain at the 48-week follow-up within the treatment (−1.19; 95% CI, −1.92 to −0.46; *p* < 0.001) and control groups (−1.52; 95% CI, −2.23 to −0.80; *p* < 0.001), pain over 24 h at the primary end point within the treatment (−1.39; 95% CI, −2.12 to −0.66; *p* < 0.001) and control groups (−1.59; 95% CI, −2.30 to −0.87; *p* < 0.001), and pain over 7 days at the primary end point within the treatment (−1.58; 95% CI, −2.28 to −0.87; *p* < 0.001) and control groups (−1.87; 95% CI, −2.56 to −1.19; *p* < 0.001).

### 3.2. Secondary Outcomes

#### 3.2.1. Depression Score

At the primary end point, there was no significant difference in the change in depression scores between the treatment and control groups (3.39; 95% CI, −0.45 to 7.22; *p* = 0.11).

There was a significant difference in the depression score at the primary end point in the control group (−2.07; 95% CI, −3.35 to −0.79 *p* < 0.001) but not for the treatment group (−0.50; 95% CI, −1.85 to 0.85 *p* = 0.88).

#### 3.2.2. Treatment Expectation

There was no significant difference in the Global Impression of Change between the treatment and control groups at the 6-week follow-up from baseline (−0.20; 95% CI, −0.92 to 0.52 *p* = 0.89) or any other time (see Appendix B).

There were also no significant differences in the Global Impression of Change from baseline at the 6-week follow up within the treatment (0.07; 95% CI, −0.00 to 0.13 *p* = 0.06), and control groups (0.03; 95% CI, −0.03 to 0.10 *p* = 0.55) or at any other time (see Appendix B).

## 4. Discussion

The fear-avoidance model offers a cognitive–behavioral explanation for the transition from acute musculoskeletal pain to chronic low back pain (cLBP) and disability [37,38,39,40]. In this framework, catastrophic pain appraisals amplify fear of movement (kinesiophobia), prompting avoidance behaviors that contribute to deconditioning, negative affect, and persistent disability. In contrast, individuals with more optimistic appraisals are less likely to avoid activity, remain engaged in valued life goals, and show higher likelihood of recovery. While there was no difference between the treatment and control groups, both groups had a clinically significant improvement in pain and disability. Given the fact that the population in this study had a back pain history of 10.6 (10.2) years, the clinically meaningful and sustained improvement in pain and disability shown here is likely not due to the simple effect of time [41] or placebo [42]. More likely, both groups benefitted from the graded activity they were exposed to in the *Dodgeality* VR games.

The main aims of this study were to explore the effects of two levels of graded exposure therapy, applied with the VR *Dodgeality* games, on patients with cLBP and fear of movement. We did not see any differences between the treatment group (increased graded exposure, i.e., balls targeted to elicit 15–60 degrees of lumbar flexion) and the control group (increased graded exposure, i.e., balls targeted to elicit 0–30 degrees of lumbar flexion) in pain, disability, fear measures (TSK and PCS), lumbar flexion angles, and the Global Impression of Change. However, we did see clinically meaningful improvements in pain, disability, fear of movement, pain catastrophizing, and the Global Impression of Change within both groups [43,44]. This indicates that both groups not only perceived they received treatment, but the maintenance of the treatment gain also suggests that both treatments were effective graded activity treatments for cLBP. Where previous VR treatment studies showed initial improvement post-treatment, they did not show improvements in pain and disability beyond 6 months, indicating that the VR was helpful in distracting from the pain [11,13] but not in treating the underling mechanisms. These findings align with reports that graded activity and exposure produce modest long-term gains, with recurrence rates of up to 41% at 12 months [10], yet extend prior work by demonstrating sustained benefits with a VR-based format [6]. VR may offer distinct advantages—immersive distraction from pain, goal-oriented engagement, and progressive challenge—which can enhance adherence and counter the attrition typical of conventional protocols [8,45,46]. Participants with high fear of movement described the VR dodgeball task as enjoyable and intrinsically motivating [18], underscoring its potential to reshape pain-related beliefs and behaviors.

Additionally, we expected the lumbar flexion excursion to increase post-treatment. However, pain catastrophizing and kinesiophobia decreased post-treatment, but the lumbar flexion excursions remained unchanged. It is known that movement restriction often persists despite cognitive improvement [14]. We expected that motor patterns would normalize within the 48-week follow-up window [47], especially with the maintained reduction in pain, harm expectancy, kinesiophobia, and pain catastrophizing measure. In previous studies, we have shown lumbar excursions around 20° to a target eliciting 40° and around 30° to a target eliciting 60° in people without back pain [48] and around 7° and 17° to similarly placed targets in people with back pain [30]. One explanation of the lack of improvement could be that participants in this study had lumbar flexion angles of 27° and 31° to targets targeting 45° and 60°, respectively, which are comparable to the lumbar flexion angles reported in people without back pain [48]. Additionally, reduced excursions were more pronounced in participants with a higher age or BMI, reflecting known declines in spinal mobility from approximately age 40 [6] and diminished flexion in individuals with obesity [7]. Given the sample’s mean age (40) and BMI (30), these effects were expected. Notably, the lumbar mobility did not influence the pain, fear, or depression outcomes; only disability scores on the Roland–Morris Disability Questionnaire correlated with the BMI, possibly reflecting functional limitations attributable to body size rather than pain alone. See Appendix A: Mixed effect model outcome table.

Limitations in this clinical trial were that the control group still received a graded exposure treatment targeting lumbar flexion. This prevented us from showing that VR graded exposure treatment targeting movements people with cLBP avoid, such as lumbar flexion, is more effective than cognitive behavioral or less specific graded activity treatments. In future studies, we suggest comparing graded exposure and activity treatments targeting lumbar flexion movements in people with cLBP compared to a treatment that does not involve lumbar flexion movements. Additionally, the targeted lumbar flexion range differed (15–60° vs. −15–30°), and completion of either protocol did not require achieving these ranges, potentially diluting any differential treatment effect. The sample size was smaller than originally planned due to recruitment challenges during the COVID-19 pandemic, which may have reduced the statistical power to detect between-group differences. These factors should be considered when interpreting the findings and underscore the need for larger-scale studies employing more distinct treatment conditions.

Collectively, these results support VR-mediated graded exposure specifically targeting lumbar motion as a promising adjunct for cLBP with high pain-related fear. By combining cognitive distraction, affective reward, and graduated physical challenge, VR can address both psychological and behavioral components of the fear–avoidance cycle, offering a scalable approach to enhance adherence and sustain recovery.

## 5. Conclusions

This study demonstrates that nine weeks of VR-based graded activity, delivered through a virtual dodgeball game, produced clinically meaningful reductions in pain and disability in individuals with cLBP and high pain-related fear, likely not due to the simple effect of time [41] or placebo [42], with benefits maintained for up to 48 weeks. These outcomes occurred without measurable increases in lumbar flexion during standardized reach tasks and were not influenced by age or BMI. VR engagement was associated with reductions in pain catastrophizing and kinesiophobia, despite persistent movement restriction, suggesting that cognitive shifts may precede or occur independently of biomechanical changes.

The VIGOR findings highlight VR’s potential to integrate cognitive distraction, affective reward, and progressive physical challenge in a format that sustains adherence and enhances enjoyment compared to conventional graded exposure/activity programs. By enabling repeated graded exposure to lumbar motion in a non-threatening game-based environment, VR interventions may help recalibrate threat perceptions, promote confidence in movement, and support the generalization of these benefits into daily life, offering a promising adjunct for disrupting the fear–avoidance cycle in cLBP.

## Figures and Tables

**Table 2:** Schedule of assessments. * indicates assessment at the first visit of each week.

	Pre-Screen	Screen	Enrollment and Baseline	Treatment	Post-Treatment Assessments	Post-Treatment Assessments
Assessment		Visit 0	Visit 1	Visits 2–19	Visit 20	Visits 21–24
Numeric Pain Rating Scales 7-day and 24 h	x	x	x	x *	x	x
Roland–Morris Disability Questionnaire	x	x	x		x	x
Fear Question	x	x				
Medical History—Back Pain	x	x				
Tampa Scale for Kinesiophobia	x	x	x		x	x
Medication Log		x	x	x	x	x
Informed Consent Form		x				
Inclusion/Exclusion Criteria		x				
Enrollment/Randomization		x				
Medical History		x				
Physical Exam Form		x				
Adverse Events			x	x	x	x
Numeric Pain Rating Scale—Right Now			x	x	x	x
Standardized Reaching Paradigm Pain and Harm Expectancy Lumbar Spine Flexion			x	x *	x	x
Center for Epidemiologic Studies—Depression			x		x	x
Pain Catastrophizing Scale			x		x	x
Pain Resilience Scale			x		x	x
Pain Self Efficacy Questionnaire			x		x	x
Brief Pain Inventory—Short Form			x		x	x
PROMIS-Anxiety			x		x	x
PROMIS-Depression			x		x	x
PROMIS-Positive Affect			x		x	x
PROMIS—Meaning and Purpose Scale			x		x	x
Life Fulfillment Scale			x		x	x
Profile of Mood States			x		x	x
Real World Activity Monitoring			x		x	x
Treatment Evaluation Inventory—Short Form			x		x	
Patient Global Impression of Change					x	x

x indicates the visit number assessments were recorded.

**Table 3:** Game version played per session per week.

Game	Treatment Session								
	Week 1	Week 2	Week 3	Week 4	Week 5	Week 6	Week 7	Week 8	Week 9
Dodgeball Cannon	2, 3, 4								
Dodgeball Day		5, 6, 7	8						
Dodgeball Night			9, 10	11, 12	13				
Dodgeball Space					14	15, 16	17	18	19

**Table 4:** Participant characteristics.

	Treatment	Control
	(N = 74)	(N = 80)
Age		
Mean (SD)	38.4 (11.9)	40.9 (13.0)
Missing	1 (1.4%)	1 (1.3%)
Sex		
Male	23 (31.1%)	30 (37.5%)
Female	51 (68.9%)	50 (62.5%)
Body mass index		
Mean (SD)	30.6 (7.57)	29.9 (8.56)
Missing	1 (1.4%)	0 (0%)
Low back pain history in years (SD)	10.8 (10.6)	10.3 (9.9)
Radiating pain		
Yes	52 (70.3%)	51 (63.8%)
No	22 (29.7%)	29 (36.3%)
>70%, per-protocol analysis		
Yes	55 (74.3%)	61 (76.3%)
No	19 (25.7%)	19 (23.8%)

**Table 5:** The self-report measures for each group at pre-treatment and at the five post-treatment assessment intervals.

Outcome	Group	Weeks Post-Treatment					
		Pre-Treatment	1	6	12	24	48
Current Pain	Treatment	4.7 (1.9)	2.9 (2.0)	3.2 (2.1)	3.2 (2.3)	3.0 (2.4)	2.9 (2.4)
	Control	4.6 (2.0)	2.8 (2.4)	2.6 (2.2)	2.5 (2.0)	2.6 (2.3)	2.6 (2.5)
Pain (24 h)	Treatment	5.4 (1.9)	3.3 (1.9)	3.6 (2.3)	3.7 (2.5)	3.6 (2.6)	3.3 (2.5)
	Control	5.0 (2.1)	3.3 (2.5)	2.9 (2.2)	2.9 (2.2)	2.7 (2.1)	3.0 (2.5)
Pain (7 days)	Treatment	5.7 (1.8)	3.6 (2.1)	4.0 (2.6)	3.5 (2.5)	3.8 (2.4)	3.4 (2.2)
	Control	5.7 (1.9)	3.3 (2.3)	3.2 (2.2)	3.3 (2.4)	3.1 (2.2)	3.1 (2.4)
Roland–Morris Disability Score	Treatment	12.8 (5.9)	9.4 (6.2)	8.4 (6.8)	8.0 (7.1)	7.1 (6.5)	7.8 (7.6)
	Control	13.5 (4.9)	8.5 (5.9)	8.2 (6.2)	7.9 (6.3)	7.2 (6.4)	6.1 (6.7)
Tampa Scale for Kinesiophobia	Treatment	43.3 (6.0)	39.5 (7.4)	38.4 (6.8)	38.2 (7.6)	37.5 (6.7)	36.5 (9.3)
	Control	43.1 (5.8)	37.4 (5.9)	37.4 (6.4)	36.9 (7.3)	36.9 (6.7)	36.6 (7.7)
Depression Score	Treatment	17.1 (8.8)	16.1 (9.7)	15.9 (10.4)	15.8 (10.6)	15.7 (12.4)	15.4 (10.5)
	Control	16.0 (9.9)	12.1 (8.9)	11.7 (8.2)	11.8 (9.9)	12.3 (10.3)	11.7 (9.6)
Pain Catastrophizing Score	Treatment	21.9 (11.9)	14.1 (12.6)	14.6 (12.8)	14.3 (14.2)	12.8 (12.6)	13.4 (13.1)
	Control	19.8 (10.6)	14.8 (10.4)	14.5 (10.9)	13.2 (11.0)	12.4 (10.0)	11.6 (11.4)
Global Impression of Change	Treatment		2.5 (1.6)	2.4 (2.0)	2.6 (1.8)	2.6 (2.0)	3.0 (1.6)
	Control		2.9 (1.4)	2.6 (2.2)	2.6 (2.0)	2.7 (1.9)	3.0 (2.1)

## Data Availability

The original data presented in this study are available on request from vanderveens24@ecu.edu due to size of the time series data (4 TB of space). The technical challenges of loading this in a public data base are limited.

## Abbreviations

The following abbreviations are used in this manuscript:

VIGOR	Virtual immersive gaming to optimize recovery
VR	Virtual reality
BMI	Body mass index
cLBP	Chronic low back pain
LBP	Low back pain

## Appendix A

**Table A1:** Linear mixed effect model fixed effect coefficient estimates by outcome.

Outcome	Term	Estimate	95% Confidence Interval	*p*-Value
Current Pain	Treatment	0.0319	[−0.6464, 0.7102]	0.9263
	Week	−0.1192	[−0.1485, −0.0899]	<0.001
	Week^2^	0.0016	[0.0011, 0.0021]	<0.001
	Male	−0.6656	[−1.2861, −0.0450]	0.0357
	Age	0.0101	[−0.0140, 0.0342]	0.4082
	BMI	0.0043	[−0.0331, 0.0417]	0.8201
	Radiating Pain	−0.0665	[−0.6927, 0.5597]	0.8341
	Treatment/Week Interaction	0.0328	[−0.0097, 0.0753]	0.1306
	Treatment/Week^2^ Interaction	−0.0005	[−0.0012, 0.0002]	0.1896
Pain (24 h)	Treatment	0.2061	[−0.4843, 0.8965]	0.5571
	Week	−0.1316	[−0.1610, −0.1022]	<0.001
	Week^2^	0.0018	[0.0013, 0.0023]	<0.001
	Male	−0.7153	[−1.3505, −0.0802]	0.0276
	Age	0.0105	[−0.0141, 0.0352]	0.4006
	BMI	−0.0010	[−0.0392, 0.0373]	0.9600
	Radiating Pain	−0.1028	[−0.7436, 0.5379]	0.7516
	Treatment/Week Interaction	0.0339	[−0.0088, 0.0766]	0.1193
	Treatment/Week^2^ Interaction	−0.0005	[−0.0012, 0.0002]	0.1415
Pain (7 days)	Treatment	0.0401	[−0.6532, 0.7334]	0.9093
	Week	−0.1424	[−0.1707, −0.1140]	<0.001
	Week^2^	0.0019	[0.0014, 0.0024]	<0.001
	Male	−0.6534	[−1.3010, −0.0059]	0.0480
	Age	0.0086	[−0.0165, 0.0338]	0.4987
	BMI	0.0004	[−0.0385, 0.0393]	0.9848
	Radiating Pain	0.0018	[−0.6512, 0.6548]	0.9957
	Treatment/Week Interaction	0.0383	[−0.0028, 0.0794]	0.0677
	Treatment/Week^2^ Interaction	−0.0006	[−0.0013, 0.0001]	0.0969
Roland–Morris Disability Score	Treatment	−0.1940	[−2.0767, 1.6888]	0.8392
	Week	−0.3106	[−0.3738, −0.2473]	<0.001
	Week^2^	0.0037	[0.0026, 0.0047]	<0.001
	Male	−1.5637	[−3.4099, 0.2825]	0.0963
	Age	0.0677	[−0.0039, 0.1394]	0.0636
	BMI	0.1356	[0.0252, 0.2459]	0.0164
	Radiating Pain	−1.4316	[−3.2902, 0.4270]	0.1301
	Treatment/Week Interaction	0.0035	[−0.0884, 0.0953]	0.9405
	Treatment/Week^2^ Interaction	0.0003	[−0.0013, 0.0018]	0.7359
Tampa Scale for Kinesiophobia	Treatment	0.8634	[−1.2259, 2.9526]	0.4163
	Week	−0.3687	[−0.4463, −0.2910]	<0.001
	Week^2^	0.0049	[0.0036, 0.0061]	<0.001
	Male	1.6859	[−0.3184, 3.6903]	0.0986
	Age	0.0341	[−0.0436, 0.1119]	0.3871
	BMI	−0.0136	[−0.1337, 0.1065]	0.8228
	Radiating Pain	−1.1620	[−3.1812, 0.8572]	0.2574
	Treatment/Week Interaction	0.0518	[−0.0611, 0.1646]	0.3681
	Treatment/Week^2^ Interaction	−0.0012	[−0.0031, 0.0006]	0.1937
Depression Score	Treatment	1.8141	[−1.2402, 4.8684]	0.2430
	Week	−0.2383	[−0.3496, −0.1271]	<0.001
	Week^2^	0.0031	[0.0013, 0.0050]	<0.001
	Male	1.1206	[−1.8220, 4.0631]	0.4529
	Age	−0.0551	[−0.1692, 0.0590]	0.3416
	BMI	0.0154	[−0.1608, 0.1915]	0.8635
	Radiating Pain	−1.3366	[−4.2997, 1.6265]	0.3742
	Treatment/Week Interaction	0.1834	[0.0217, 0.3452]	0.0263
	Treatment/Week^2^ Interaction	−0.0026	[−0.0053, 0.0001]	0.0550
Pain Catastrophizing Score	Treatment	2.1451	[−1.4669, 5.7570]	0.2431
	Week	−0.3551	[−0.4854, −0.2248]	<0.001
	Week^2^	0.0040	[0.0018, 0.0062]	<0.001
	Male	0.4956	[−2.9951, 3.9862]	0.7794
	Age	0.0789	[−0.0565, 0.2143]	0.2513
	BMI	−0.0043	[−0.2133, 0.2047]	0.9677
	Radiating Pain	−3.9321	[−7.4473, −0.4168]	0.0286
	Treatment/Week Interaction	−0.1596	[−0.3488, 0.0296]	0.0981
	Treatment/Week^2^ Interaction	0.0026	[−0.0005, 0.0058]	0.0995
Global Impression of Change	Treatment	−0.2295	[−0.8281, 0.3692]	0.4500
	Week	0.0066	[−0.0038, 0.0170]	0.2119
	Male	0.1248	[−0.4479, 0.6975]	0.6665
	Age	0.0024	[−0.0198, 0.0246]	0.8306
	BMI	0.0169	[−0.0187, 0.0526]	0.3487
	Radiating Pain	0.0990	[−0.4863, 0.6843]	0.7380
	Treatment/Week Interaction	0.0064	[−0.0083, 0.0212]	0.3915
Left Lumbar Flexion (lvl 1)	Treatment	−0.6638	[−3.3326, 2.0050]	0.6245
	Week	0.0289	[−0.0737, 0.1314]	0.5803
	Male	−1.9796	[−4.4424, 0.4832]	0.1142
	Age	−0.0470	[−0.1424, 0.0483]	0.3308
	BMI	−0.1387	[−0.2900, 0.0126]	0.0720
	Radiating Pain	1.3049	[−1.1804, 3.7903]	0.3009
	Treatment/Week Interaction	−0.0084	[−0.1553, 0.1385]	0.9107
Right Lumbar Flexion (lvl 1)	Treatment	−1.2747	[−3.6308, 1.0814]	0.2877
	Week	−0.0402	[−0.1392, 0.0589]	0.4257
	Male	−1.9656	[−4.0524, 0.1213]	0.0647
	Age	−0.0532	[−0.1340, 0.0275]	0.1945
	BMI	−0.0642	[−0.1930, 0.0646]	0.3258
	Radiating Pain	0.4404	[−1.6657, 2.5466]	0.6798
	Treatment/Week Interaction	0.0680	[−0.0740, 0.2101]	0.3466
Left Lumbar Flexion (lvl 2)	Treatment	−1.2736	[−5.4183, 2.8711]	0.5456
	Week	0.0713	[−0.2242, 0.3667]	0.6356
	Week^2^	−0.0023	[−0.0108, 0.0063]	0.6008
	Male	−0.0270	[−3.7430, 3.6890]	0.9885
	Age	−0.2022	[−0.3455, −0.0589]	0.0061
	BMI	−0.3904	[−0.6177, −0.1632]	<0.001
	Radiating Pain	2.4387	[−1.2937, 6.1710]	0.1984
	Treatment/Week Interaction	0.3495	[−0.0708, 0.7699]	0.1029
	Treatment/Week^2^ Interaction	−0.0097	[−0.0220, 0.0027]	0.1252
Right Lumbar Flexion (lvl 2)	Treatment	−1.3265	[−5.0614, 2.4084]	0.4845
	Week	−0.0536	[−0.1465, 0.0394]	0.2579
	Male	−0.1351	[−3.7278, 3.4576]	0.9408
	Age	−0.1901	[−0.3285, −0.0516]	0.0075
	BMI	−0.3558	[−0.5749, −0.1367]	0.0017
	Radiating Pain	1.9967	[−1.6093, 5.6027]	0.2754
	Treatment/Week Interaction	0.0752	[−0.0604, 0.2108]	0.2760
Left Lumbar Flexion (lvl 3)	Treatment	−0.8817	[−5.7198, 3.9563]	0.7197
	Week	−0.0227	[−0.1431, 0.0978]	0.7116
	Male	2.1445	[−2.4639, 6.7530]	0.3588
	Age	−0.2407	[−0.4179, −0.0634]	0.0082
	BMI	−0.4637	[−0.7450, −0.1825]	0.0014
	Radiating Pain	2.2588	[−2.3552, 6.8727]	0.3345
	Treatment/Week Interaction	0.0961	[−0.0771, 0.2693]	0.2758
Right Lumbar Flexion (lvl 3)	Treatment	−1.3970	[−6.0022, 3.2083]	0.5503
	Week	−0.0959	[−0.2059, 0.0141]	0.0872
	Male	2.1380	[−2.3281, 6.6040]	0.3453
	Age	−0.2551	[−0.4274, −0.0828]	0.0040
	BMI	−0.4941	[−0.7677, −0.2206]	<0.001
	Radiating Pain	1.7435	[−2.7390, 6.2260]	0.4429
	Treatment/Week Interaction	0.1134	[−0.0463, 0.2731]	0.1635
Left Lumbar Flexion (lvl 4)	Treatment	−1.3593	[−6.4115, 3.6929]	0.5963
	Week	0.0383	[−0.0888, 0.1654]	0.5540
	Male	3.5048	[−1.3170, 8.3266]	0.1528
	Age	−0.3120	[−0.4982, −0.1258]	0.0012
	BMI	−0.4993	[−0.7931, −0.2056]	0.0010
	Radiating Pain	2.2680	[−2.5793, 7.1153]	0.3563
	Treatment/Week Interaction	0.0112	[−0.1742, 0.1965]	0.9057
Right Lumbar Flexion (lvl 4)	Treatment	−0.1181	[−5.2082, 4.9720]	0.9636
	Week	−0.0209	[−0.1483, 0.1065]	0.7474
	Male	3.5237	[−1.3810, 8.4284]	0.1576
	Age	−0.2966	[−0.4857, −0.1076]	0.0024
	BMI	−0.4690	[−0.7694, −0.1686]	0.0025
	Radiating Pain	2.6439	[−2.2779, 7.5657]	0.2899
	Treatment/Week Interaction	−0.0050	[−0.1882, 0.1782]	0.9575

## Appendix B

**Table A2:** Change in outcome from baseline by treatment group and week post-treatment.

Outcome	Week Post-Treatment	Change from Baseline *				Difference in Change from Baseline (Trt vs. Ctrl) *	
		Treatment		Control			
		Estimate [95% CI]	*p*-Value	Estimate [95% CI]	*p*-Value	Estimate [95% CI]	*p*-Value
Current Pain	1	−0.75 [−1.10, −0.39]	<0.001	−1.03 [−1.37, −0.69]	<0.001	0.31 [−0.51, 1.13]	0.8640
	6	−1.04 [−1.52, −0.55]	<0.001	−1.42 [−1.88, −0.96]	<0.001	0.42 [−0.43, 1.26]	0.6873
	12	−1.31 [−1.91, −0.71]	<0.001	−1.79 [−2.35, −1.22]	<0.001	0.51 [−0.38, 1.40]	0.5401
	24	−3.21 [−4.17, −2.24]	<0.001	−3.77 [−5.03, −2.52]	<0.001	0.60 [−0.35, 1.55]	0.4378
	48	−1.19 [−1.92, −0.46]	<0.001	−1.52 [−2.23, −0.80]	<0.001	0.36 [−0.77, 1.49]	0.9314
Pain (24 h)	1	−0.85 [−1.21, −0.49]	<0.001	−1.13 [−1.47, −0.80]	<0.001	0.49 [−0.35, 1.33]	0.5114
	6	−1.18 [−1.66, −0.69]	<0.001	−1.56 [−2.03, −1.10]	<0.001	0.59 [−0.27, 1.46]	0.3357
	12	−1.49 [−2.09, −0.88]	<0.001	−1.96 [−2.53, −1.39]	<0.001	0.68 [−0.23, 1.59]	0.2488
	24	−3.62 [−4.60, −2.65]	<0.001	−4.16 [−5.42, −2.90]	<0.001	0.74 [−0.22, 1.71]	0.2248
	48	−1.39 [−2.12, −0.66]	<0.001	−1.59 [−2.30, −0.87]	<0.001	0.40 [−0.74, 1.55]	0.8957
Pain (7 days)	1	−0.91 [−1.25, −0.56]	<0.001	−1.23 [−1.56, −0.91]	<0.001	0.37 [−0.49, 1.22]	0.7945
	6	−1.26 [−1.73, −0.79]	<0.001	−1.70 [−2.15, −1.26]	<0.001	0.48 [−0.39, 1.36]	0.5753
	12	−1.59 [−2.17, −1.02]	<0.001	−2.14 [−2.69, −1.59]	<0.001	0.59 [−0.33, 1.51]	0.4161
	24	−3.87 [−4.81, −2.94]	<0.001	−4.51 [−5.72, −3.29]	<0.001	0.67 [−0.30, 1.65]	0.3315
	48	−1.58 [−2.28, −0.87]	<0.001	−1.87 [−2.56, −1.19]	<0.001	0.34 [−0.79, 1.47]	0.9453
Roland–Morris Disability Score	1	−2.68 [−3.44, −1.91]	<0.001	−2.74 [−3.46, −2.01]	<0.001	−0.13 [−2.53, 2.27]	1.0000
	6	−3.72 [−4.76, −2.68]	<0.001	−3.83 [−4.82, −2.84]	<0.001	−0.08 [−2.53, 2.37]	1.0000
	12	−4.71 [−6.00, −3.42]	<0.001	−4.90 [−6.12, −3.68]	<0.001	−0.01 [−2.54, 2.53]	1.0000
	24	−9.48 [−11.56, −7.40]	<0.001	−9.88 [−12.58, −7.18]	<0.001	0.21 [−2.43, 2.84]	0.9999
	48	−4.71 [−6.25, −3.16]	<0.001	−5.75 [−7.27, −4.24]	<0.001	0.85 [−2.05, 3.76]	0.9486
Tampa Scale for Kinesiophobia	1	−2.81 [−3.75, −1.86]	<0.001	−3.20 [−4.09, −2.31]	<0.001	1.26 [−1.36, 3.88]	0.7077
	6	−3.94 [−5.23, −2.65]	<0.001	−4.44 [−5.66, −3.22]	<0.001	1.36 [−1.33, 4.05]	0.6590
	12	−5.06 [−6.65, −3.47]	<0.001	−5.60 [−7.10, −4.10]	<0.001	1.40 [−1.40, 4.20]	0.6687
	24	−11.32 [−13.89, −8.76]	<0.001	−11.68 [−15.01, −8.35]	<0.001	1.22 [−1.72, 4.16]	0.8132
	48	−6.33 [−8.24, −4.42]	<0.001	−5.25 [−7.12, −3.38]	<0.001	−0.22 [−3.53, 3.09]	1.0000
Depression Score	1	−0.50 [−1.85, 0.85]	0.8782	−2.07 [−3.35, −0.79]	<0.001	3.39 [−0.45, 7.22]	0.1129
	6	−0.71 [−2.55, 1.13]	0.8586	−2.87 [−4.61, −1.13]	<0.001	3.98 [0.05, 7.90]	0.0467
	12	−0.92 [−3.20, 1.35]	0.8282	−3.62 [−5.77, −1.47]	<0.001	4.51 [0.42, 8.60]	0.0225
	24	−4.36 [−8.02, −0.69]	0.0107	−7.55 [−12.31, −2.79]	<0.001	5.01 [0.73, 9.29]	0.0128
	48	−1.44 [−4.17, 1.30]	0.6268	−3.36 [−6.03, −0.70]	0.0056	3.74 [−1.06, 8.55]	0.2104
Pain Catastrophizing Score	1	−4.48 [−6.06, −2.91]	<0.001	−3.15 [−4.65, −1.66]	<0.001	0.81 [−3.74, 5.36]	0.9943
	6	−6.23 [−8.38, −4.07]	<0.001	−4.42 [−6.46, −2.39]	<0.001	0.34 [−4.31, 5.00]	0.9999
	12	−7.88 [−10.54, −5.22]	<0.001	−5.69 [−8.21, −3.17]	<0.001	−0.04 [−4.88, 4.80]	1.0000
	24	−13.71 [−18.00, −9.42]	<0.001	−11.32 [−16.89, −5.74]	<0.001	−0.25 [−5.31, 4.82]	1.0000
	48	−7.75 [−10.97, −4.54]	<0.001	−7.22 [−10.35, −4.10]	<0.001	1.62 [−4.06, 7.30]	0.9554
Global Impression of Change	6	0.07 [−0.00, 0.13]	0.0559	0.03 [−0.03, 0.10]	0.5487	−0.20 [−0.92, 0.52]	0.8873
	12	0.14 [−0.00, 0.29]	0.0560	0.07 [−0.07, 0.22]	0.5487	−0.16 [−0.85, 0.54]	0.9310
	24	0.30 [−0.01, 0.60]	0.0559	0.15 [−0.15, 0.45]	0.5487	−0.08 [−0.78, 0.62]	0.9899
	48	0.61 [−0.01, 1.24]	0.0557	0.31 [−0.30, 0.92]	0.5487	0.07 [−0.82, 0.97]	0.9964
Left Lumbar Flexion (lvl 1)	1	0.20 [−1.12, 1.53]	0.9761	0.29 [−1.00, 1.58]	0.9328	−0.75 [−3.69, 2.20]	0.9050
	6	0.31 [−1.68, 2.30]	0.9761	0.43 [−1.51, 2.37]	0.9328	−0.79 [−3.94, 2.36]	0.9084
	12	0.43 [−2.35, 3.21]	0.9761	0.61 [−2.11, 3.32]	0.9328	−0.84 [−4.54, 2.86]	0.9298
	24	0.68 [−3.70, 5.05]	0.9761	0.95 [−3.31, 5.22]	0.9328	−0.94 [−6.29, 4.41]	0.9655
Right Lumbar Flexion (lvl 1)	1	0.28 [−1.01, 1.56]	0.9379	−0.40 [−1.65, 0.85]	0.8269	−0.59 [−3.09, 1.91]	0.9205
	6	0.42 [−1.51, 2.35]	0.9379	−0.60 [−2.48, 1.27]	0.8269	−0.25 [−2.97, 2.46]	0.9945
	12	0.59 [−2.11, 3.28]	0.9379	−0.84 [−3.47, 1.78]	0.8269	0.15 [−3.14, 3.45]	0.9993
	24	0.92 [−3.32, 5.16]	0.9379	−1.33 [−5.45, 2.80]	0.8269	0.97 [−4.00, 5.94]	0.9536
Left Lumbar Flexion (lvl 2)	1	3.01 [0.03, 6.00]	0.0472	0.48 [−2.49, 3.46]	0.9961	1.26 [−3.90, 6.41]	0.9762
	6	3.62 [−0.07, 7.32]	0.0575	0.56 [−3.14, 4.26]	0.9974	1.79 [−3.62, 7.21]	0.9153
	12	3.57 [−0.43, 7.57]	0.1058	0.49 [−3.53, 4.52]	0.9990	1.80 [−3.70, 7.31]	0.9191
	24	3.13 [−9.40, 15.67]	0.9733	2.12 [−10.17, 14.42]	0.9949	−0.27 [−7.08, 6.55]	1.0000
Right Lumbar Flexion (lvl 2)	1	0.22 [−1.03, 1.46]	0.9671	−0.54 [−1.71, 0.64]	0.6237	−0.57 [−4.85, 3.70]	0.9843
	6	0.32 [−1.55, 2.19]	0.9671	−0.80 [−2.56, 0.96]	0.6237	−0.20 [−4.51, 4.11]	0.9993
	12	0.45 [−2.16, 3.07]	0.9671	−1.12 [−3.59, 1.34]	0.6237	0.25 [−4.31, 4.82]	0.9989
	24	0.71 [−3.40, 4.83]	0.9671	−1.77 [−5.64, 2.10]	0.6237	1.16 [−4.48, 6.79]	0.9476
Left Lumbar Flexion (lvl 3)	1	0.73 [−0.84, 2.31]	0.6065	−0.23 [−1.75, 1.30]	0.9789	0.08 [−5.42, 5.58]	1.0000
	6	1.10 [−1.26, 3.46]	0.6064	−0.34 [−2.62, 1.94]	0.9789	0.56 [−4.96, 6.08]	0.9931
	12	1.54 [−1.76, 4.85]	0.6064	−0.48 [−3.67, 2.72]	0.9789	1.14 [−4.68, 6.95]	0.9542
	24	2.42 [−2.77, 7.61]	0.6064	−0.75 [−5.77, 4.28]	0.9789	2.29 [−4.84, 9.42]	0.8294
Right Lumbar Flexion (lvl 3)	1	0.17 [−1.29, 1.64]	0.9888	−0.96 [−2.35, 0.43]	0.2715	−0.26 [−5.58, 5.06]	0.9992
	6	0.26 [−1.93, 2.45]	0.9888	−1.44 [−3.52, 0.64]	0.2715	0.30 [−5.06, 5.66]	0.9988
	12	0.37 [−2.70, 3.44]	0.9888	−2.01 [−4.93, 0.90]	0.2715	0.98 [−4.66, 6.63]	0.9668
	24	0.58 [−4.25, 5.40]	0.9888	−3.17 [−7.75, 1.42]	0.2715	2.35 [−4.51, 9.21]	0.8011
Left Lumbar Flexion (lvl 4)	1	0.49 [−1.21, 2.20]	0.8679	0.38 [−1.22, 1.99]	0.9211	−1.25 [−6.99, 4.50]	0.9387
	6	0.74 [−1.82, 3.30]	0.8679	0.57 [−1.84, 2.98]	0.9211	−1.19 [−6.96, 4.58]	0.9467
	12	1.04 [−2.54, 4.62]	0.8679	0.80 [−2.57, 4.18]	0.9211	−1.12 [−7.24, 4.99]	0.9613
	24	1.63 [−4.00, 7.26]	0.8679	1.26 [−4.04, 6.56]	0.9211	−0.99 [−8.56, 6.58]	0.9854
Right Lumbar Flexion (lvl 4)	1	−0.26 [−1.92, 1.40]	0.9760	−0.21 [−1.82, 1.40]	0.9857	−0.17 [−6.01, 5.68]	0.9998
	6	−0.39 [−2.88, 2.11]	0.9760	−0.31 [−2.73, 2.10]	0.9857	−0.19 [−6.09, 5.70]	0.9998
	12	−0.54 [−4.03, 2.95]	0.9760	−0.44 [−3.82, 2.94]	0.9857	−0.22 [−6.47, 6.02]	0.9997
	24	−0.85 [−6.34, 4.63]	0.9760	−0.69 [−6.00, 4.62]	0.9857	−0.28 [−7.96, 7.40]	0.9997

* Baseline was measured at one week post-treatment for Global Impression of Change and at pre-treatment for all other outcomes.
